# Long-Term High Dietary Diversity Maintains Good Physical Function in Chinese Elderly: A Cohort Study Based on CLHLS from 2011 to 2018

**DOI:** 10.3390/nu14091730

**Published:** 2022-04-21

**Authors:** Sumiya Aihemaitijiang, Li Zhang, Chen Ye, Mairepaiti Halimulati, Xiaojie Huang, Ruoyu Wang, Zhaofeng Zhang

**Affiliations:** 1 Department of Nutrition & Food Hygiene, School of Public Health, Peking University Health Science Center, Haidian District, Beijing 100191, China; 1410606101@pku.edu.cn (S.A.); 1510306235@pku.edu.cn (C.Y.); 2011210145@bjmu.edu.cn (M.H.); 13161518166@163.com (X.H.); wry7582568@163.com (R.W.); 2 Department of Population Health Sciences, School of Population Health & Environmental Sciences, King’s College London, London SE1 1UL, UK; li.1.zhang@kcl.ac.uk

**Keywords:** the elderly, physical function, basic activities of daily living, instrumental activities of daily living, dietary diversity, dietary pattern

## Abstract

(1) Objective: This study aimed to explore the correlation between dietary factors and physical function in Chinese elderly. (2) Methods: A cohort study was conducted on the association of long-term dietary intake status with physical function in older people based on the Chinese Longitudinal Healthy Longevity Survey (CLHLS) from 2011 to 2018. The physical function of the subjects was judged according to the scores of basic activities of daily living (BADL) and instrumental activities of daily living (IADL). The dietary diversity score was established according to the intake frequency of the food groups, and the dietary pattern score was obtained by factor analysis. The associations between dietary factors and functional impairment was investigated by logistic regressions. (3) Results: A total of 2282 subjects were included in our cohort study, 458 and 1439 of whom had BADL limitation and IADL limitation, respectively. The risk of functional impairment decreased in the consistent high dietary diversity groups compared with the consistent low dietary diversity group (*p* < 0.05). The fruit-egg-milk pattern, vegetable-meat-fish pattern, and condiment and tea pattern reduced the risk of functional impairment (*p* < 0.05). (4) Conclusions: Long-term maintenance of high dietary diversity and increasing total dietary intake can help maintain good physical function of Chinese elderly.

## 1. Introduction

With the development of the societal economy and the improvement of medical standards, life expectancy has been increasing, which directly leads to the rapid aging of the global population. According to one report [1], the world’s elderly aged 60 and over reached 901 million in 2015, an increase of 48% over the world in 2000. It is expected that the proportion of the world’s elderly population will reach 2.1 billion by 2030, accounting for 21.93% of the world’s total population [2]. At present, China has increasingly developed into an aging society, with 264 million people aged 60 years, of which 190 million are aged 65 years and over [3], accounting for 18.70% and 13.50% of the total population, respectively, while the number of people aged 65 years or over is expected to rise to approximately 240 million by 2030 [4].

With the aging process, the physiological and immune functions of the elderly gradually decline. Compared with middle-aged and young people, the physical health of the elderly becomes worse and worse, and the impairment of physical function is the most important health problem of the elderly [5]. Impaired physical function is closely associated with various adverse health outcomes, such as osteoporosis, falls, and fractures [6,7,8]. At the same time, it leads to frailness, loss of independence, high mortality, and low quality of life [9,10,11], which will place a serious burden on China’s medical and health expenditure [12]. Therefore, the State Council of China issued the outline of the “Healthy China 2030” plan in 2016, which pointed out the need to achieve healthy aging. In order to achieve it, it is more important to focus on the physical function proposed by the World Health Organization (WHO) as the best indicator to measure the health status of the elderly than to focus on a certain disease [13,14] because the process of physical impairment is neither inevitable nor irreversible [15] but can be intervened upon. Therefore, finding modifiable factors that can slow down, stop, or even reverse impairment and effectively control and maintain physical function are important factors to promote healthy aging [16].

It is well-known that nutrition is a key determinant of health and well-being throughout the lifecycle [17,18]. Studies have shown that high dietary quality in the elderly is associated with better health outcomes, such as the reduction of all-cause mortality, cardiovascular disease, cancer, type 2 diabetes, and neurodegenerative diseases [17,19,20]. Degenerative changes and metabolic decline in the elderly make them more sensitive to nutrient deficiency and increase their risk of malnutrition. Inadequate dietary intake is associated with many adverse health outcomes, such as impaired immune response [21], reduced antioxidant defense [22], frail [23], osteoporotic fractures [24], peripheral artery disease [25], and chronic, non-communicable diseases [26]. Optimal nutrition is important to prevent adverse health outcomes. It not only prolongs life expectancy but also improves the quality of life of the elderly, which ultimately contributes to healthy aging [27].

As an important influencing factor that can be regulated, dietary nutrition intervention is one of the main strategies for healthy aging [28,29]. However, to date, only a few studies have explored the effects of dietary diversity or dietary patterns on physical function [30,31,32,33,34], and their conclusions are not completely consistent; almost all of them focus on Western populations. What kind of diet is more suitable for the Chinese elderly is a question worth exploring. Furthermore, these studies were limited by the cross-sectional design, only using cross-sectional dietary factors, and could not observe the impact of long-term dietary habits, the high compliance of a high-quality diet, or changes in dietary factors. This study conducted a cohort study using Chinese Longitudinal Healthy Longevity Survey (CLHLS) data from 2011 to 2018 to explore the correlation between dietary factors and physical function of the elderly and put forward reasonable dietary suggestions to prevent or slow down the physical function impairment of the Chinese elderly and help them with healthy aging.

## 2. Materials and Methods

### 2.1. Data Collection and Samples

**Figure 1:**
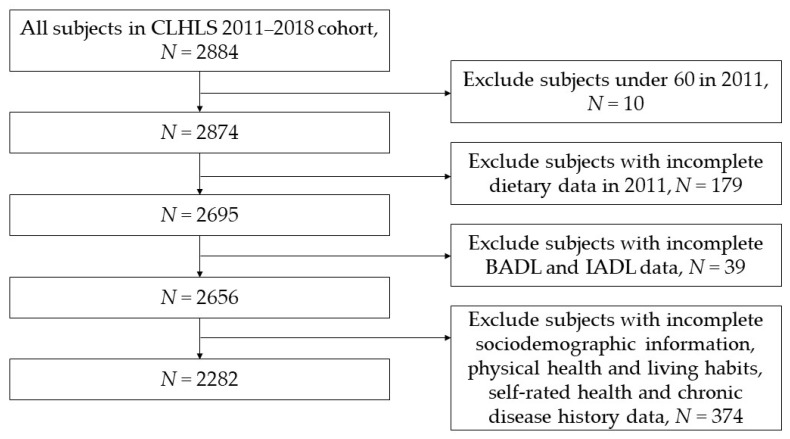
Flow chart for inclusion and exclusion of research subjects.

This cohort study is based on the CLHLS, which is a follow-up survey of elderly individuals organized by the Research Center for Healthy Aging and Development of Peking University/National Development Research Institute. The survey covers 23 provinces in China. The questionnaires were divided into two types: the surviving respondents’ questionnaire and the families of the dead elderly questionnaire. After the baseline survey in 1998, the project conducted follow-up surveys in 2000, 2002, 2005, 2008–2009, 2011–2012, 2014, and 2017–2018, 

In this study, subjects in CLHLS who were followed in both 2011 and 2018 were included in the cohort. The research content included dietary-related data, basic activities of daily living (BADL), instrumental activities of daily living (IADL), sociodemographic information, physical health and living habits, self-rated health, and some chronic disease history of the subjects in the 2011–2018 cohort [35]. According to the inclusion and exclusion criteria in Figure 1, a total of 2282 subjects were finally included in this study.

All subjects gave their informed consent for inclusion before they participated in the study. The study was approved by the biomedical ethics committee of Peking University (IRB00001052–24713074).

### 2.2. Dietary Diversity and Dietary Pattern Assessment

#### 2.2.1. Dietary Diversity Assessment

This study applied dietary diversity score (DDS) to evaluate the initial dietary diversity in 2011. The intake frequency in the CLHLS survey was used to indicate the intake status of food groups, including fresh fruit, vegetable, meat, fish, eggs, bean products, salt-preserved vegetables, sugar, garlic, milk products, nut products, mushrooms or algae, and tea. The DDS was calculated according to the intake frequency of 13 food groups. The specific intake frequency and scoring criteria are shown in Table A1.

The total DDS is the sum of the scores of 13 food groups, with the lowest score being 0 and the highest score being 13. The higher the score, the better the dietary diversity. According to the score, the level of dietary diversity was divided into two groups: low DDS (<7) and high DDS (≥7).

We also expressed changes in dietary diversity (CDD) from 2011 to 2018 using four categories: consistently low dietary diversity (low-low), consistently high dietary diversity (high-high), and inconsistent dietary diversity (low-high, high-low) (Table A2).

#### 2.2.2. Dietary Pattern Assessment

In this study, the principal component analysis method of exploratory factor analysis was used to extract the dietary patterns of the elderly population in CLHLS research. The number of factors was determined according to the results of factor analysis of dietary intake in 2011, such as eigenvalues, gravel maps, and interpretability of each factor. We explained and named the model according to professional knowledge and reserved food or food groups.

The pattern score was calculated based on the intake frequency of each food and its weight (factor score). The higher the score of the diet pattern, the greater the preference for a specific diet pattern. According to the dietary pattern score in 2011, the subjects were divided into low- to high-quartile groups (Q1, Q2, Q3, and Q4).

### 2.3. Definition of Physical Function

Based on the availability of CLHLS survey data and the physiological health characteristics of the elderly, BADL scale and IADL scale were used to evaluate the physical function of the elderly in 2018.

BADL assessed by the Katz index [36] was used to evaluate physical function, which includes 6 basic abilities: bathing, dressing, toileting, indoor transferring, continence, and feeding. BADL is the sum of the scores of six indicators, and the total score varies between 0 (lack of performance) and 6 (maximum performance). According to the BADL, the subjects were divided into no-limitations in BADL group (BADL score = 6) and limitations in BADL group (BADL score = 0–5) (Table A3).

IADL was assessed with the IADL scale [37], which includes the scores of 8 items: visiting outside, shopping outside, cooking independently, washing independently, walking continuously, lifting heavy objects continuously, squatting continuously, and taking transportation independently. IADL is the sum of the scores of eight indicators, and the total score varies between 0 (lack of performance) and 8 (maximum performance). According to the IADL, the subjects were divided into no-limitations in IADL group (IADL score = 8) and limitations in IADL group (IADL score = 0–7) (Table A3).

### 2.4. Assessment of Covariates

According to previous studies [30,31,32,33], sociodemographic information (gender, age, residence, living conditions, and education), physical health and living habits (body mass index (BMI), alcohol consumption, smoking status, and exercise), other dietary factors (staple food, main flavor, and vitamins intake), and self-rated health and history of chronic diseases (hypertension, diabetes, heart disease, stroke or cardiovascular disease (CVD), arthritis, rheumatism or rheumatoid disease, dyslipidemia) were taken as covariates of this study.

### 2.5. Statistical Analysis

Independent chi-square tests were used to examine the initial basic characteristics of the groups, such as gender, age, residence, living conditions, education, BMI, alcohol consumption, smoking status, exercise, staple food intake, main flavor, vitamins intake, self-rated health, and history of chronic diseases.

We constructed multilevel logistic regression models to account for potential confounders. They were mainly analyzed by five models: Model 1 was a multiple logistic regression model of dietary factors and functional impairment without any adjustment. Model 2 was adjusted according to sociodemographic information. Model 3 was adjusted according to sociodemographic information and physical health and living habits. Model 4 was adjusted according to sociodemographic information, physical health and living habits, and other dietary factors. Model 5 was adjusted according to sociodemographic information, physical health and living habits, other dietary factors, self-rated health, and history of chronic diseases.

Data preprocessing, database establishment, and statistical analysis were all completed by SAS University edition software (Copyright © 2012–2020, SAS Institute Inc., Cary, NC, USA) unless otherwise indicated. *p* < 0.05 was used as the statistical index of significance test.

## 3. Results

### 3.1. Baseline Characteristics

A total of 458 subjects were limited by BADL of the 2282 subjects, while 1439 subjects were limited by IADL. 

It was found that gender, age, residence, living conditions, education, BMI, alcohol consumption, smoking status, exercise, staple food intake, main flavor, self-rated health, history of hypertension, history of stroke or cerebrovascular disease, and history of rheumatism or rheumatoid disease were the influencing factors of BADL limitation (*p* < 0.05) (Table 1).

It was found that gender, age, education, BMI, alcohol consumption, smoking status, exercise, self-rated health, history of stroke or cerebrovascular disease, history of arthritis, history of rheumatism or rheumatoid disease were the influencing factors of IADL limitation (*p* < 0.05) (Table 2).

### 3.2. The Association of Dietary Diversity and Physical Function

Taking whether the physical function (BADL and IADL) was limited as the dependent variable and DDS in 2011 with CDD as the independent variable, multiple logistic regression analysis was conducted.

Through the BADL model, it was found that dietary diversity had no effect on the occurrence of BADL limitation. Through IADL model 1, it was found that compared with high dietary diversity, the odds ratio (OR) value of subjects with low dietary diversity for IADL limitation was 1.283 (95% confidence intervals (CI): 1.080–1.525), indicating that subjects with low dietary diversity had an increased risk of IADL restriction than subjects with high dietary diversity. However, after the adjustment of models 2, 3, 4, and 5, dietary diversity had no effect on the occurrence of IADL limitation (Table 3).

Through the BADL model, it was found that CDD had no effect on the occurrence of BADL limitation. Through IADL model 1, it was found that compared with consistently low dietary diversity, the OR value of subjects with consistently high dietary diversity and dietary diversity that became progressively better for IADL limitation was 0.512 (95%CI: 0.408–0.642) and 0.602 (95%CI: 0.472–0.768), indicating that subjects with consistently high dietary diversity and dietary diversity that became better had a lower risk of IADL limitation than those who with consistently low dietary diversity. After the adjustment of models 2, 3, 4, and 5, the impact still exists (*p* < 0.05) (Table 4).

### 3.3. The Association of Dietary Pattern and Physical Function

The applicability test result of exploratory factor analysis was KMO = 0.806 > 0.8, indicating that the above foods or food groups were suitable for principal component analysis. The results of Bartlett’s test of sphericity showed that χ^2^ = 3572.241 (*p* < 0.0001), indicating that the food groups were not independent of each other and had a strong correlation, so factor analysis could be carried out. According to Kaiser criteria, this study decided to retain three factors. The eigenvalues of the three factors after rotation were 2.278, 1.631, and 1.554, respectively, and the cumulative contribution rate was 42.024% (Table A4 and Table A5).

Through factor analysis, three dietary patterns were obtained. According to the food characteristics of each pattern, they were named fruit-egg-milk pattern (fresh fruit, eggs, milk products, nut products, and mushroom or algae), vegetable-meat-fish pattern (vegetable, meat, and fish), and condiment and tea pattern (salt-preserved vegetable, sugar, garlic, and tea) (Table A6).

Taking whether the physical function (BADL and IADL) was limited as the dependent variable and dietary pattern score in 2011 as the independent variable, multiple logistic regression analysis was conducted.

Through BADL model 1 and 2, it was found that compared with the subjects with the lowest quantile array of fruit-egg-milk pattern score, the subjects with the highest, third, and second quantiles of this pattern score had a lower risk of BADL limitation after 7 years (*p* < 0.05). Compared with the subjects in the lowest quantile array of vegetable-meat-fish pattern scores, the subjects in the highest, third, and second quantiles of this pattern score had a lower risk of BADL limitation after 7 years (*p* < 0.05). However, there was no correlation in condiment and tea pattern with BADL limitation (Table 5).

Through IADL model 1 and 2, it was found that compared with the subjects with the lowest quantile array of fruit-egg-milk pattern score, the subjects with the highest and second quantile of this pattern score had a lower risk of IADL limitation after 7 years (*p* < 0.05). Compared with the subjects in the lowest quantile array of vegetable-meat-fish pattern score, the subjects in the highest, third, and second quantiles of this pattern score had a lower risk of IADL limitation after 7 years (*p* < 0.05). Compared with the subjects in the lowest quantile array of condiment and tea pattern score, the subjects in the highest and third quantile of this pattern score had a lower risk of IADL limitation after 7 years (*p* < 0.05).

## 4. Discussion

Dietary diversity has always been considered by nutritionists as an important factor to improve dietary quality [38]. By increasing dietary diversity, we can ensure sufficient nutrient intake and improve dietary quality so as to improve the nutritional status of the body and promote health [39]. This study used cohort data from 2011 to 2018 in CLHLS to evaluate the impact of dietary factors on the physical function of Chinese elderly. We found that long-term high dietary diversity and increasing the total intake maintains physical function.

In this study, there was no significant correlation between the initial dietary diversity and physical function after 7 years. A cross-sectional study in Japan [30] found that dietary diversity was closely related to the physical function of the elderly in the community. Some cross-sectional studies have found that [40,41,42] there was a correlation between dietary quality and the occurrence of physical function limitation. The results of this study are not consistent with those of previous studies. This may be related to the fact that the effect of the initial cross-sectional diet in our study was not sufficiently detectable, while over a cumulative period of seven years, the effect was sufficiently enough to be detectable.

Because diet is a long-term accumulation process, the impact of long-term dietary changes or long-term eating habits on physical function should be explored. Therefore, we investigated the impact of CDD on physical function from 2011 to 2018. Our study found that long-term maintenance of high dietary diversity was conducive to maintaining physical function in the Chinese elderly after adjusting for gender, age, residence, living condition, education, BMI, alcohol consumption, smoking status and exercise, self-rated health, and chronic disease history (stroke and CVD, arthritis, rheumatism or rheumatoid disease), which is similar to the results of a longitudinal study [31], which reported a 50% lower risk of impaired IADLs after 5 years in the highest quartile of diet assessed by the modified Australian diet quality index compared to the lowest quartile in 895 Australian elderly. We also found that even if the initial dietary diversity was not good, improving dietary diversity was beneficial to maintaining physical function. This meant that as long as dietary diversity was increased, it would help to maintain the physical function of the elderly, which is more practical based on the fact that it is difficult to maintain high dietary diversity for the elderly.

The internal relationship between dietary diversity and physical function may have the following explanations: first, the research showed [43] that the nutritional adequacy of diet could be predicted by calculating the number of food groups, which meant the greater the number of food groups, the richer the dietary diversity, and the more adequate the nutrition intake of the human body, which was positively helpful for the elderly to maintain better physical function. Secondly, the role of many nutrients in the body was affected by the existence and balance of other nutrients at the same time [44]. Therefore, only when there was rich dietary diversity, multiple nutrients could reach a balanced state, and the interaction of multiple nutrients could better promote the health of the body. For example, the intake of vitamin D, vitamin C, and vitamin K2 can help the body better absorb calcium [45] so as to protect physical function.

In order to explore which diet was more beneficial to maintain the physical function of the elderly, we further used exploratory factor analysis for dimensionality reduction. Three dietary patterns were found: fruit-egg-milk pattern, vegetable-meat-fish pattern, and condiment and tea pattern. Our study showed that the score of fruit-egg-milk pattern, vegetable-meat-fish pattern, and condiment and tea pattern were negatively correlated with the occurrence of physical function limitation after 7 years, which indicates that increasing the total dietary intake was the protective factor for the occurrence of physical function limitation. Our result was similar to the other results. Two cross-sectional studies [46,47] found an association between higher compliance with the Mediterranean diet pattern and better physical function. Another longitudinal study [48] found that for women, a healthy Nordic diet predicted better physical performance at 10-year follow-up. All three dietary pattern scores were negatively correlated with the occurrence of physical function limitation, possibly due to the prevalence of malnutrition among the elderly in China. Therefore, the elderly should be encouraged to intake more. No matter what kind of dietary pattern, more intake could better ensure energy intake and nutritional intake, which was more critical. However, it did not mean that each dietary pattern was the same. Although there were no more findings in this study, we still suggest that the elderly eat more favorable foods, such as high-quality protein, good fats, and a Mediterranean-style diet.

The aging of the elderly is multifactorial and the mechanism is very complex, which may be due to insufficient protein, chronic inflammation, and insufficient vitamins and minerals. It is beneficial for the elderly to supplement any food from different dimensions. The internal relationship between dietary patterns and physical function may be explained in the following ways: First, meat, fish, eggs, bean products, and milk products were the main sources of high-quality protein. Dietary protein was mainly responsible for muscle protein metabolism in the elderly [42], which was related to less muscle loss [49] and less impairment of physical function [40]. This suggests that adequate protein intake helps the elderly maintain good muscle function [50]. Second, milk products were the main source of calcium and vitamin D, which could reduce the risk of impaired physical function associated with osteoporosis, osteoporosis-related fractures, and decreased muscle strength [41]. Third, inflammation and oxidative stress were considered to be one of the main causes of aging, accelerating the loss of muscle and bone mass [51,52]. Fresh fruits, vegetable, mushroom, algae, tea, and garlic were important sources of antioxidants (such as vitamin C and carotene), which were related to enhancing skeletal muscle strength. Adequate intake could reduce impairment of physical function by reducing inflammatory response and oxidative stress [53]. Therefore, the combination of these nutrients with the food group could partially explain the protective association between dietary diversity, adequate intake, and physical function.

One of the strengths of this study was that our study was a 7-year cohort study, and we observed the effects of long-term dietary maintenance or dietary changes on physical function and not just a cross-sectional dietary intake. Second, the data come from CLHLS, covering 23 provinces or municipalities. These areas were different in geographical location, economic development level, public resources, and health indicators and were more representative of Chinese residents.

Several methodological limitations should be considered. The self-reported dietary intake frequency through the food frequency questionnaire was prone to recall bias. In addition, the questionnaire only had food intake frequency without food intake. The frequency might not completely match the total intake. There were only large food groups, which was not detailed enough. Finally, although we adjusted for many potential confounding factors and considered covariant changes, we cannot rule out the effects of residual and unmeasured confounding in this observational study.

## 5. Conclusions

In conclusion, long-term maintenance of high dietary diversity and increasing total dietary intake can help maintain good physical function of Chinese elderly. A good diet is never too late. Even if the previous dietary status is not good, as long as the elderly improve their dietary status, it will be helpful. This study provides a good evidence that a scientific diet is conducive to promoting healthy aging.

## Figures and Tables

**Table 1:** Baseline characteristics of BADL non-limitation group and BADL limitation group (*n* = 2282).

Variable	BADL Non-Limitation (*n*, %)	BADL Limitation (*n*, %)	χ^2^	*p*
Gender			39.8553	<0.0001 *
Male	958 (85.31)	165 (14.69)		
Female	866 (74.72)	293 (25.28)		
Age			179.0328	<0.0001 *
≥60 and <80	1246 (88.81)	157 (11.19)		
≥80	578 (65.76)	301 (34.24)		
Residence			7.7287	0.0054 *
Urban	891 (77.61)	257 (22.39)		
Rural	933 (82.28)	201 (17.72)		
Living conditions			19.7802	<0.0001 *
Alone	372 (87.74)	52 (12.26)		
Not alone	1452 (78.15)	406 (21.85)		
Education			43.1407	<0.0001 *
0 year	809 (74.22)	281 (25.78)		
1–6 years	715 (84.52)	131 (15.48)		
≥7 years	300 (86.71)	46 (13.29)		
BMI			13.8744	0.0010 *
<18.5	266 (75.35)	87 (24.65)		
≥18.5 and <25	1228 (75.86)	266 (24.14)		
≥25	330 (82.20)	105 (17.80)		
Alcohol consumption			5.6969	0.0170 *
No	1055 (78.26)	293 (21.74)		
Yes	769 (82.33)	165 (17.67)		
Smoking status			12.6830	0.0004 *
No	1052 (77.47)	306 (22.53)		
Yes	772 (83.55)	152 (16.45)		
Exercise			41.1264	<0.0001 *
No	1180 (76.23)	368 (23.77)		
Yes	644 (87.74)	90 (12.26)		
Staple food			35.9080	<0.0001 *
Rice	1137 (84.04)	216 (15.96)		
Corn (maize)	49 (70.00)	21 (30.00)		
Wheat (noodles and bread, etc.)	375 (74.85)	126 (25.15)		
Rice and wheat	263 (73.46)	95 (26.54)		
Main flavor			17.8833	0.0031 *
Insipidity	1254 (79.12)	331 (20.88)		
Salty	359 (85.27)	62 (14.73)		
Sweet	93 (70.99)	38 (29.01)		
Hot	43 (89.58)	5 (10.42)		
Crude	3 (75.00)	1 (25.00)		
Do not have all the above tastes	72 (77.42)	21 (22.58)		
Vitamins intake			1.9730	0.1601
Not often	1631 (80.34)	399 (19.66)		
Often	193 (76.59)	59 (23.41)		
Self-rated health			52.7000	<0.0001 *
Very bad	19 (54.29)	16 (45.71)		
Bad	185 (66.79)	92 (33.21)		
So-so	703 (80.80)	167 (19.20)		
Good	697 (83.47)	138 (16.53)		
Very good	220 (83.02)	45 (16.98)		
Hypertension			4.8824	0.0271 *
No	1035 (78.35)	789 (21.65)		
Yes	286 (82.10)	172 (17.90)		
Diabetes			0.3229	0.5699
No	1599 (80.11)	397 (19.89)		
Yes	225 (78.67)	61 (21.33)		
Heart disease			0.0753	0.7837
No	1468 (80.48)	366 (19.96)		
Yes	356 (79.46)	92 (20.54)		
Stroke or CVD			19.5695	<0.0001 *
No	1590 (81.45)	362 (18.55)		
Yes	234 (70.91)	96 (29.09)		
Arthritis			0.0157	0.9004
No	1581 (19.89)	398 (20.11)		
Yes	243 (80.20)	60 (19.80)		
Rheumatism or rheumatoid disease			3.9248	0.0476 *
No	1664 (80.46)	404 (19.54)		
Yes	160 (74.77)	54 (25.23)		
Dyslipidemia			0.0106	0.9179
No	1686 (79.91)	424 (20.09)		
Yes	138 (80.23)	34 (19.77)		

*: *p* < 0.05.

**Table 2:** Baseline characteristics of IADL non-limitation group and IADL limitation group (*n* = 2282).

Variable	IADL Non-Limitation (*n*, %)	IADL Limitation (*n*, %)	χ^2^	*p*
Gender			99.7973	<0.0001 *
Male	530 (47.20)	593 (52.80)		
Female	313 (27.01)	846 (72.99)		
Age			320.0123	<0.0001 *
≥60 and <80	719 (51.25)	684 (48.75)		
≥80	124 (14.11)	755 (85.89)		
Residence			0.1256	0.7230
Urban	420 (36.59)	728 (63.41)		
Rural	423 (37.30)	711 (62.70)		
Living conditions			0.8709	0.3507
Alone	165 (38.92)	259 (61.08)		
Not alone	678 (36.49)	1180 (63.51)		
Education			160.7759	<0.0001 *
0 year	268 (24.59)	822 (75.41)		
1–6 years	371 (43.85)	475 (56.15)		
≥7 years	204 (58.96)	142 (41.04)		
BMI			16.1147	0.0003 *
<18.5	97 (27.48)	256 (72.52)		
≥18.5 and <25	580 (38.16)	914 (61.84)		
≥25	166 (38.82)	269 (61.18)		
Alcohol consumption			20.2127	<0.0001 *
No	447 (33.16)	901 (66.84)		
Yes	396 (42.40)	538 (57.60)		
Smoking status			32.6432	<0.0001 *
No	437 (32.18)	921 (67.82)		
Yes	406 (43.94)	518 (56.06)		
Exercise			94.7844	<0.0001 *
No	467 (30.17)	1081 (69.83)		
Yes	376 (51.23)	358 (48.77)		
Staple food			0.8819	0.8298
Rice	503 (37.18)	850 (62.82)		
Corn (maize)	23 (32.86)	47 (67.14)		
Wheat (noodles and bread, etc.)	189 (37.72)	312 (62.28)		
Rice and wheat	128 (35.75)	230 (64.25)		
Main flavor			7.0127	0.2197
Insipidity	579 (36.53)	1006 (63.47)		
Salty	159 (37.77)	262 (62.23)		
Sweet	43 (32.82)	88 (67.18)		
Hot	23 (47.92)	25 (52.08)		
Crude	0 (0.00)	4 (100.0)		
Do not have all the above tastes	39 (41.94)	54 (58.06)		
Vitamins intake			0.9632	0.3264
Not often	757 (37.29)	1273 (62.71)		
Often	86 (34.13)	166 (65.87)		
Self-rated health			106.8733	<0.0001 *
Very bad	5 (14.29)	30 (85.71)		
Bad	53 (19.13)	224 (80.87)		
So-so	271 (31.15)	599 (68.85)		
Good	377 (45.15)	458 (54.85)		
Very good	137 (51.70)	128 (48.30)		
Hypertension			1.5137	0.2186
No	502 (38.00)	819 (62.00)		
Yes	341 (35.48)	620 (64.52)		
Diabetes			3.1984	0.0737
No	751 (37.63)	1245 (62.37)		
Yes	92 (32.17)	194 (67.83)		
Heart disease			2.5057	0.1134
No	692 (37.73)	1142 (62.27)		
Yes	151 (33.71)	297 (66.29)		
Stroke or CVD			12.7071	0.0004 *
No	750 (38.42)	1202 (61.58)		
Yes	93 (28.18)	237 (71.82)		
Arthritis			11.8498	0.0006 *
No	758 (38.30)	1221 (61.70)		
Yes	85 (28.05)	218 (71.95)		
Rheumatism or rheumatoid disease			6.4382	0.0112 *
No	781 (37.77)	1287 (62.23)		
Yes	62 (28.97)	152 (71.03)		
Dyslipidemia			1.5342	0.2155
No	787 (37.30)	1323 (62.70)		
Yes	56 (32.56)	116 (67.44)		

*: *p* < 0.05.

**Table 3:** Logistic regression analysis between dietary diversity and physical function (*n* = 2282).

Model	Physical Function	DDS (<7:≥7)		*p*
		OR	(95 CI%)	
	BADL			
1		1.030	0.836, 1.271	0.7786
2		0.946	0.752, 1.189	0.6327
3		0.985	0.781, 1.243	0.9012
4		1.087	0.857, 1.380	0.4913
5		0.991	0.775, 1.268	0.9445
	IADL			
1		1.283	1.080, 1.525	0.0047 *
2		1.065	0.875, 1.296	0.5314
3		1.059	0.866, 1.294	0.5775
4		1.017	0.826, 1.252	0.8743

*: *p* < 0.05. Model 1, no adjustment; Model 2, adjusted for gender, age, residence, living condition, and education; Model 3, model 2 + BMI, alcohol consumption, smoking status, and exercise; Model 4, model 3 + staple food and main flavor; Model 5 = model 4 + self-rated health and chronic disease history; Model 6, model 3 + self-rated health and chronic disease history.

**Table 4:** Logistic regression analysis between change of dietary diversity and physical function (*n* = 2282).

Model	Physical Function	CDD			
		Consistently Low Dietary Diversity	Dietary Diversity Gets Worse	Dietary Diversity Gets Better	Consistently High Dietary Diversity
	BADL				
1		1	0.981 (0.747, 1.288)	0.781 (0.576, 1.060)	0.841 (0.636, 1.112)
2		1	0.990 (0.739, 1.328)	0.749 (0.542, 1.034)	0.958 (0.704, 1.303)
3		1	0.937 (0.696, 1.262)	0.790 (0.570, 1.096)	0.966 (0.706, 1.322)
4		1	0.856 (0.630, 1.162)	0.717 (0.512, 1.002)	0.812 (0.588, 1.122)
5		1	0.919 (0.671, 1.258)	0.757 (0.535, 1.072)	0.942 (0.672, 1.320)
	IADL				
1		1	0.899 (0.709, 1.140)	0.602 (0.472, 0.768) *	0.512 (0.408, 0.642) *
2		1	0.990 (0.762, 1.287)	0.592 (0.451, 0.778) *	0.651 (0.502, 0.844) *
3		1	0.971 (0.744, 1.266)	0.632 (0.479, 0.833) *	0.694 (0.532, 0.906) *
6		1	1.000 (0.760, 1.316)	0.713 (0.533, 0.954) *	0.783 (0.593, 0.994) *

*: *p* < 0.05. Model 1, no adjustment; Model 2, adjusted for gender, age, residence, living condition, and education; Model 3, model 2 + BMI, alcohol consumption, smoking status, and exercise; Model 4, model 3 + staple food and main flavor; Model 5, model 4 + self-rated health and chronic disease history; Model 6, model 3 + self-rated health and chronic disease history.

**Table 5:** Logistic regression analysis between dietary pattern score and physical function (*n* = 2282).

Model	Physical Function/Dietary Pattern	Dietary Pattern Score			
		Q1	Q2	Q3	Q4
	BADL				
1	fruit-egg-milk pattern	1	0.820 (0.616, 1.091)	0.799 (0.600, 1.064)	0.715 (0.533, 0.959) *
	vegetable-meat-fish pattern	1	0.621 (0.469, 0.823) *	0.602 (0.455, 0.797) *	0.454 (0.337, 0.610) *
	condiment and tea pattern	1	0.779 (0.586, 1.035)	0.772 (0.580, 1.028)	0.655 (0.489, 0.879) *
2	fruit-egg-milk pattern	1	0.698 (0.504, 0.967) *	0.616 (0.440, 0.863) *	0.504 (0.349, 0.720) *
	vegetable-meat-fish pattern	1	0.693 (0.502, 0.956) *	0.665 (0.479, 0.923) *	0.573 (0.405, 0.812) *
	condiment and tea pattern	1	0.746 (0.539, 1.031)	0.877 (0.632, 1.219)	0.796 (0.568, 1.117)
	IADL				
1	fruit-egg-milk pattern	1	0.765 (0.596, 0.983) *	0.772 (0.601, 0.992) *	0.594 (0.463, 0.760) *
	vegetable-meat-fish pattern	1	0.714 (0.553, 0.923) *	0.531 (0.413, 0.683) *	0.463 (0.360, 0.595) *
	condiment and tea pattern	1	0.883 (0.686, 1.138)	0.575 (0.449, 0.736) *	0.612 (0.478, 0.784) *
3	fruit-egg-milk pattern	1	0.769 (0.576, 0.966) *	0.884 (0.662, 1.182)	0.812 (0.606, 0.998) *
	vegetable-meat-fish pattern	1	0.705 (0.525, 0.948) *	0.491 (0.365, 0.659) *	0.554 (0.412, 0.744) *
	condiment and tea pattern	1	0.938 (0.700, 1.257)	0.629 (0.471, 0.839) *	0.767 (0.575, 0.993) *

*: *p* < 0.05. Model 1, no adjustment; Model 2, adjusted for gender, age, residence, living condition, education, BMI, alcohol consumption, smoking status and exercise, staple food, main flavor, self-rated health, and chronic disease history; Model 3, adjusted for gender, age, residence, living condition, education, BMI, alcohol consumption, smoking status and exercise, self-rated health, and chronic disease history.

## Data Availability

The raw data supporting the conclusions of this article can be found here: https://opendata.pku.edu.cn/dataverse/CHADS (accessed on 20 March 2022).

## Abbreviations

WHO	World Health Organization
CLHLS	Chinese Longitudinal Healthy Longevity Survey
BADL	Basic activities of daily living
IADL	Instrumental activities of daily living
BMI	Body mass index
CVD	Cardiovascular disease
DDS	Dietary diversity score
CDD	Change in dietary diversity
OR	Odds ratio
95%CI	95% confidence intervals

## Appendix A

**Table A1:** Scoring criteria for dietary diversity.

Food Group	DDS
Fresh fruit	“Every day/almost every day” = 1 “Often” = 1 “Sometimes” = 0 “Rarely or never” = 0
Fresh vegetable	
Meat	“Almost every day” = 1 “At least once a week” = 1 “At least once a month” = 0 “Occasionally” = 0 “Rarely or never” = 0
Fish	
Eggs	
Bean products	
Salt-preserved vegetable	
Sugar	
Garlic	
Milk products	
Nut products	
Mushrooms or algae	
Tea	

**Table A2:** CDD from 2011 to 2018.

CDD	DDS in 2011	DDS in 2018
Consistently low dietary diversity (low-low),	<7	<7
Dietary diversity get worse (high-low)	≥7	<7
Dietary diversity get better (low-high)	<7	≥7
Consistently high dietary diversity (high-high)	≥7	≥7

**Table A3:** Definition and score of body function.

Independent Variable	Questions	Score Assignment
BADL	1. Bathing 2. Dressing 3. Toileting 4. Indoor transferring 5. Continence 6. Feeding	Without assistance = 1 One-part assistance = 0 More than one-part assistance = 0
IADL	1. Able to go outside to visit neighbors? 2. Able to go shopping by yourself? 3. Able to make food by yourself? 4. Able to wash clothes? 5. Able to walk one kilometer? 6. Able to carry 5 kg weight? 7. Able to crouch and stand three times? 8. Able to take public transportation?	Yes = 1 A little difficult = 0 Unable to do so = 0

**Table A4:** KMO and Bartlett’s Test.

Kaiser-Meyer-Olkin Measure of Sampling Adequacy		0.806
Bartlett’s Test of Sphericity	Approx. chi-Square	3572.241
	df	78
	Sig.	<0.0001

**Table A5:** Total Variance Explained.

Component	Rotation Sums of Squared Loadings		
	Total	% of Variance	Cumulative %
1	2.278	17.524	17.524
2	1.631	12.545	30.068
3	1.554	11.955	42.024

Extraction Method: Principal Component Analysis.

**Table A6:** Dietary pattern factor load.

Food Groups	Component		
	Plant-Derived Food Pattern	Animal-Derived Food Pattern	Egg and Bean Pattern
Fresh fruit	0.551	0.361	−0.037
Vegetable	−0.033	0.507	0.165
Meat	0.093	0.716	−0.047
Fish	0.229	0.692	−0.014
Eggs	0.659	0.019	0.084
Food made from beans	0.535	0.090	0.255
Salt-preserved vegetable	−0.077	0.040	0.749
Sugar	0.291	−0.061	0.461
Garlic	0.217	0.060	0.575
Milk products	0.738	−0.041	0.017
Nut products	0.439	0.183	0.363
Mushroom or algae	0.556	0.244	0.245
Tea	0.089	0.373	0.395

Extraction Method: Principal Component Analysis. Rotation Method: Varimax with Kaiser Normalization.
